# Successful endoscopic therapy for esophagotracheal fistula using polyglycolic acid sheets

**DOI:** 10.1016/j.igie.2025.01.007

**Published:** 2025-01-17

**Authors:** Zhenkai Wang, Xiang Liu, Wanzhen Chen, Quanzhao Di, Xiaoqian Yang, Dan Liu

**Affiliations:** Department of Endoscopy Center, Nanjing Hospital of Chinese Medicine Affiliated to Nanjing University of Chinese Medicine, Nanjing, Jiangsu Province, 210004, China

A 68-year-old man with esophageal cancer developed a cough and dysphagia during radiotherapy. Gastroscopy revealed multiple tablets lodged in the esophagus, with a tracheoesophageal fistula (Figs. 1 and 2). Several tablets were then removed via gastroscopy, and the fistula opening was covered with a polyglycolic acid sheet. The surface was sprayed with autologous blood to promote the application and fixation of the polyglycolic acid sheet (Fig. 3), and a nasojejunal tube was inserted. Weekly reviews were conducted to monitor the site. If the polyglycolic acid sheet was displaced, endoscopic repositioning and reapplication of autologous blood were performed; otherwise, observation continued. At 6 weeks, gastroscopy showed healing of the esophageal fistula, and the nasojejunal tube was removed (Fig. 4). The patient was simultaneously transitioned to an open diet, which was gradually changed to a normal diet. No further symptoms, including coughing or swallowing difficulties, were reported.

**Figure 1:**
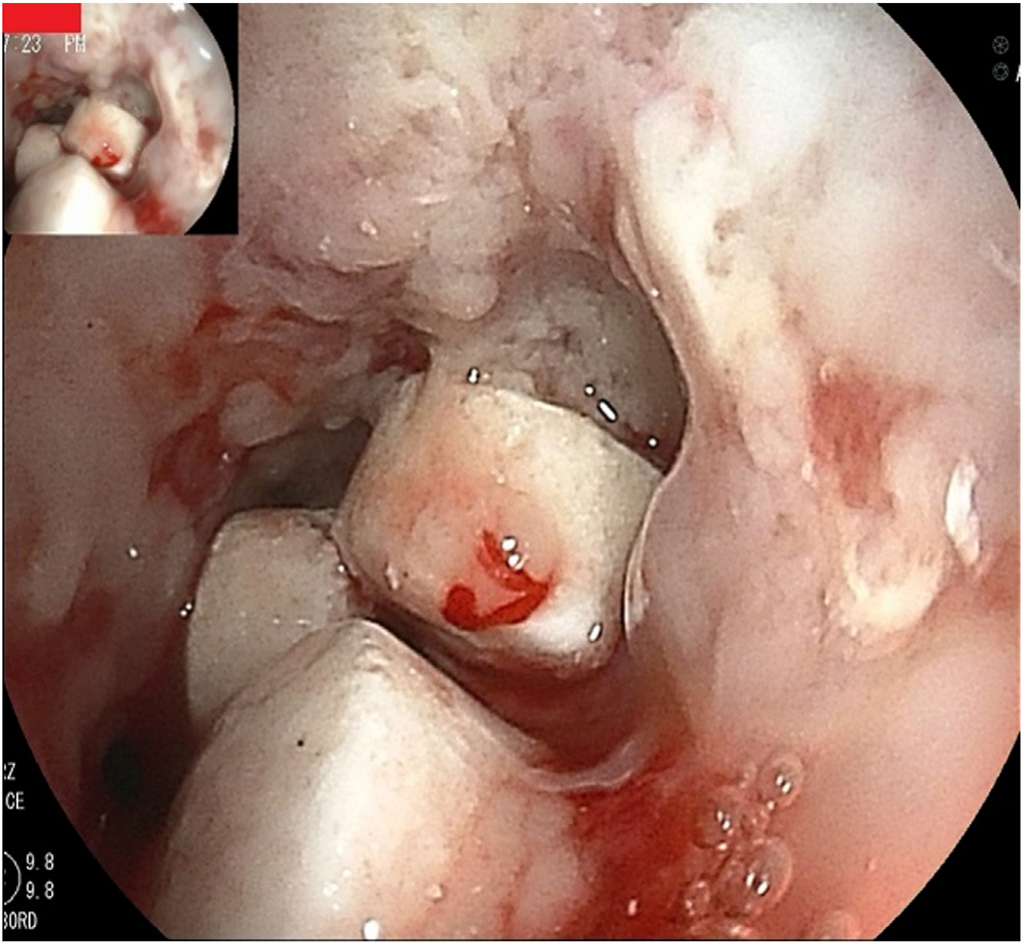
Multiple tablets (*box*) lodged in the esophagus.

**Figure 2:**
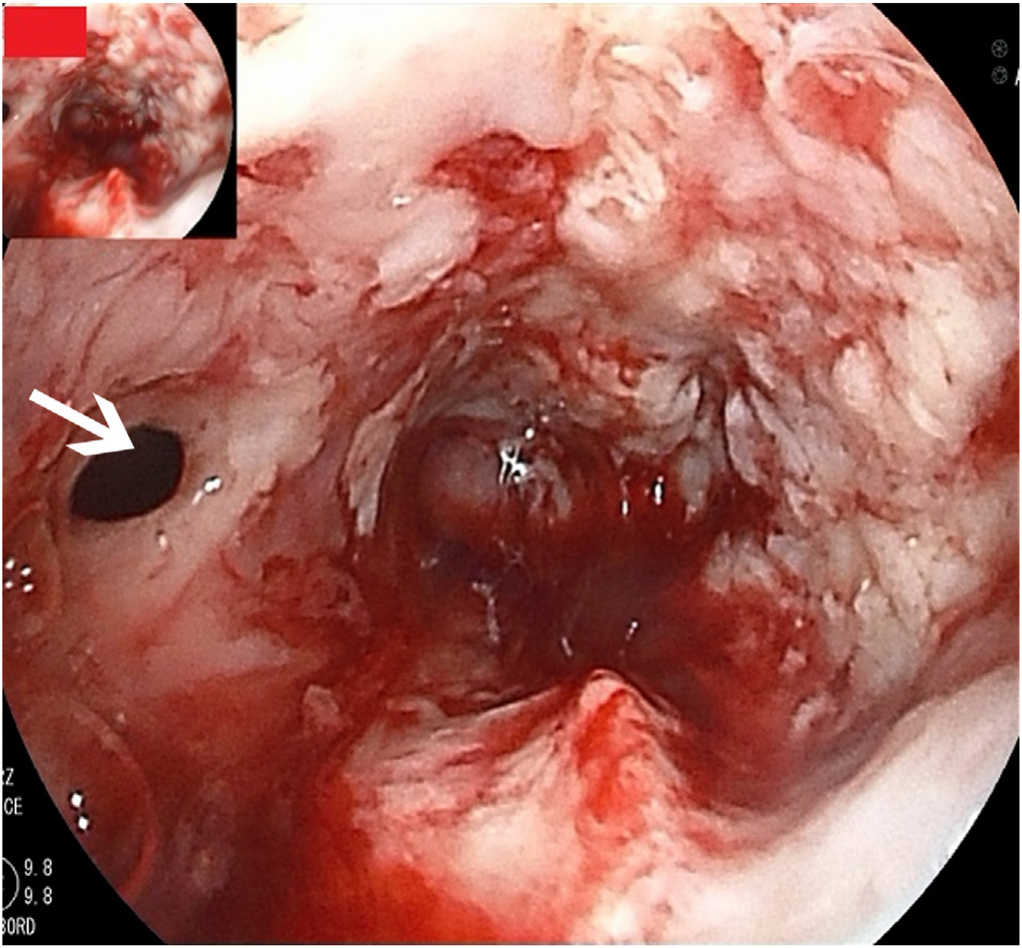
A tracheoesophageal fistula of the esophagus (*arrow*).

**Figure 3:**
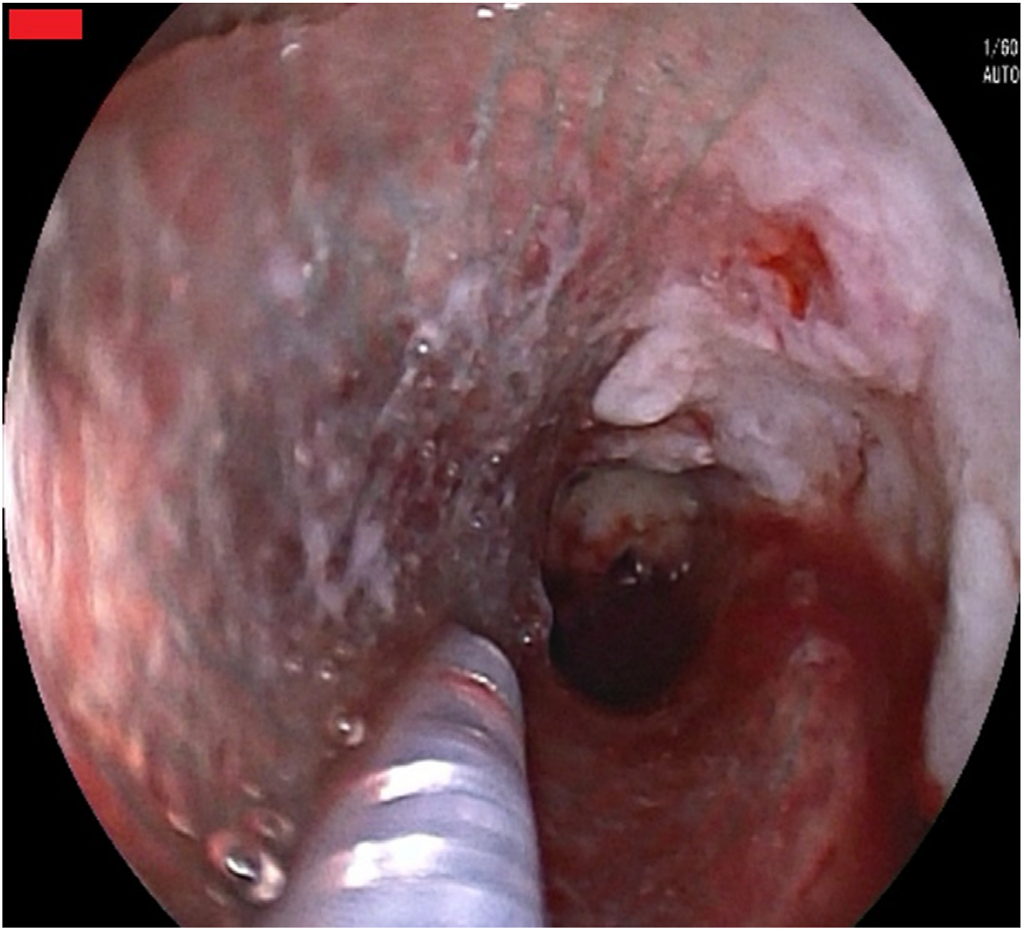
The application and fixation of the polyglycolic acid sheet.

**Figure 4:**
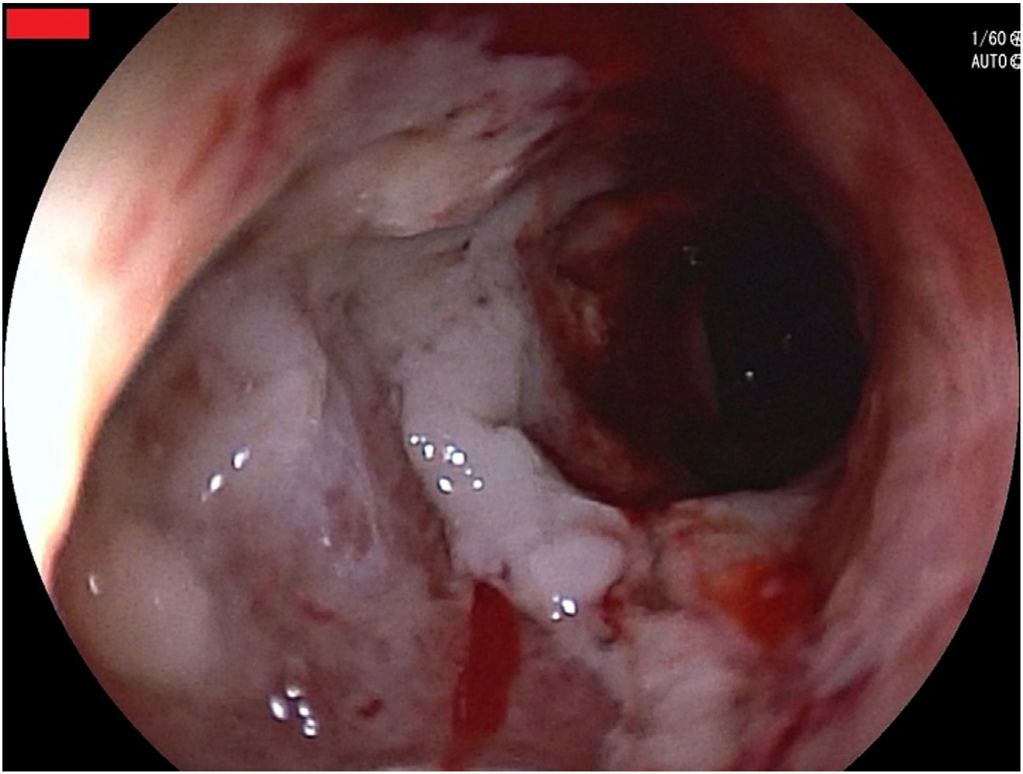
Gastroscopy showing the healing of the esophageal fistula.

Esophagotracheal fistulas due to foreign body impaction during radiotherapy are rare in patients with esophageal cancer. Considering that no standard treatment exists for such fistula, this case offers a new perspective for its treatment.

Informed consent was obtained for this case report.

## Disclosure

All authors disclosed no financial relationships.

